# The integration of sex and gender considerations in health policymaking: a scoping review

**DOI:** 10.1186/s12939-021-01411-8

**Published:** 2021-03-02

**Authors:** Allison Williams, Joonsoo Sean Lyeo, Sophie Geffros, Alexander Mouriopoulos

**Affiliations:** Hamilton, Canada

**Keywords:** Gender, Health policy, Inclusivity, Policymaking, Sex

## Abstract

While the terms ‘sex’ and ‘gender’ represent distinct concepts, their influence may intersect as important determinants of health. Despite their influence in shaping individual health outcomes, there is often inaccuracy and inconsistency in the degree to which sex and gender considerations are integrated in the health policymaking process. This primary aim of this paper is to fill the gap in the current understanding of how sex and gender considerations are integrated in this process. A scoping review methodology was used with the objective of assessing the extent to which sex and gender were considered inclusively and comprehensively in established examples of health policy planning and development. One hundred seventy-five documents from the academic and grey literature were found to meet the inclusion criteria for this scoping review. The authors charted the data from these publications, assessing the ways in which sex and gender were incorporated in their policy development process. Five key findings were ascertained from this review: (1) the terms sex and gender are often used interchangeably; (2) the terms sex and gender are often used with a limited and binary scope; (3) the most inclusive and comprehensive documents included transgender and gender diverse populations; (4) there are significant variations in the degree of inclusivity and comprehensivity of these documents based on geographic distribution; and (5) documents published within the last 5 years were more inclusive than older documents. This paper concludes with an acknowledgment of the limitations of the study design, a summary of the findings, future research directions, and implications for policymakers.

## Introduction

This scoping review is the first of three iterative stages in a wider project following the methodological framework outlined by Day et al. [1], who used this methodology in the development of essential metrics for assessing the integration of sex and gender considerations in health research proposals involving human participants. Rather than assessing sex and gender in human health research proposals, this larger project will apply the aforementioned framework in the development of essential metrics for assessing the integration of sex and gender considerations in health policymaking. It should be noted that, in this manuscript, we will be defining a ‘policy’ as a collection of principles that have been created with the intent of guiding decisions and shaping outcomes. More specifically, we will be defining ‘health policy’ as a collection of principles that have been created with the intent of guiding decisions specific to shaping health outcomes, broadly defined via the social determinants of health.

As the first of the three iterative stages, the objective of this scoping review was to determine the inclusivity and comprehensiveness of the use of sex and gender in health policymaking. We begin by providing a discussion specific to the importance of an inclusive and comprehensive understanding of sex and gender in the development and implementation of health policy. This is followed by the methods used in implementing the scoping review. Finally, the results are presented, followed by a conclusion, where next steps are outlined.

## Background

Although the terms ‘sex’ and ‘gender’ are frequently used inconsistently and interchangeably, it should be noted that they are not synonymous, and that they represent distinct concepts [2]. The term ‘sex’ refers to the genetic, physiological, and biological characteristics which have traditionally been used to distinguish males and females. This is in contrast to the term ‘gender’, which refers to the socially-constructed characteristics which have traditionally been used to distinguish men and women. It should be noted that there is a significant degree of fluidity within the concepts of sex and gender, and that current discourse has come to recognize the complexity of these concepts beyond the binary dichotomies which have traditionally been accepted in the academic literature [2]. For instance, in recognition of the fluidity of sex, there is a growing body of literature acknowledging how variations in chromosomal expression or physiological traits are not always accurately categorized into the male-female binary dichotomy, and may instead be more accurately sorted under the umbrella of ‘intersex’ variations [3].

While ‘sex’ and ‘gender’ represent distinct concepts, their influence may intersect as important, parallel determinants of health [4]. To illustrate, social determinants of health such as income, education and employment, interact with both sex and gender, leading to disparities in health status. Socio-economic factors can contribute to health inequalities, not only between women and men, but among and between different groups of men and women, shaping prospects for health outcomes [4]. For instance, the influence of gender can be seen when discussing family caregivers and caregiver burden [5]. For context, family caregivers are individuals who provide unpaid, informal caregiving services to a care recipient, typically someone in their personal network, with an underlying physical or mental health condition. Much of the literature on family caregivers is limited to the perspectives of cis men and women. That being said, current figures suggest that informal caregiving is predominantly provided by women, in part due to pervasive societal and cultural expectations regarding the role of women in family caregiving [6]. Consequently, women have been reported to spend more time providing informal caregiving, and tend to be more vulnerable to caregiving stressors (i.e. physical strain, psychosocial distress) than men.

Similarly, when looking at family caregiving through the lens of sex, variations can be seen in the experiences of male and female family caregivers [5]. For instance, female caregivers have been reported to experience a greater perception of poor health than their male counterparts. The influence of sex may not only influence the differences in the magnitude of health outcomes between male and female caregivers, but may also influence the type of outcomes displayed. For instance, when examining the sources of caregiver burden, [5] male caregivers tended to experience caregiver burden through low morale and a greater need for social support, whereas female caregivers tended to experience caregiver burden through their relationships with other family members.

Despite their importance in influencing health outcomes, there is often inconsistency in the degree to which sex and gender considerations are integrated in health policymaking. This is highlighted by factors such as: the lack of literature assessing the sex and gender dimensions of policy; the dominance of men in policymaking settings; and the presumed sex-neutral and gender-neutral stance of policies which fail to acknowledge the differential experiences of.

individuals based on their respective sex and gender [7]. This indicates that there may be a gap in our current understanding of how to best integrate sex and gender considerations in health policymaking. Assuming this to be the case, this would suggest a need for tools or strategies to guide policymakers to comprehensively integrate sex and gender in their work.

## Methods

This scoping review followed the methodological framework outlined by Arksey and O’Malley [8]. This particular methodological framework was chosen due to its well-established rigour and effectiveness [9]. Prior to beginning the scoping review, a librarian from McMaster University was consulted to assess its relevance.

**Fig. 1:**
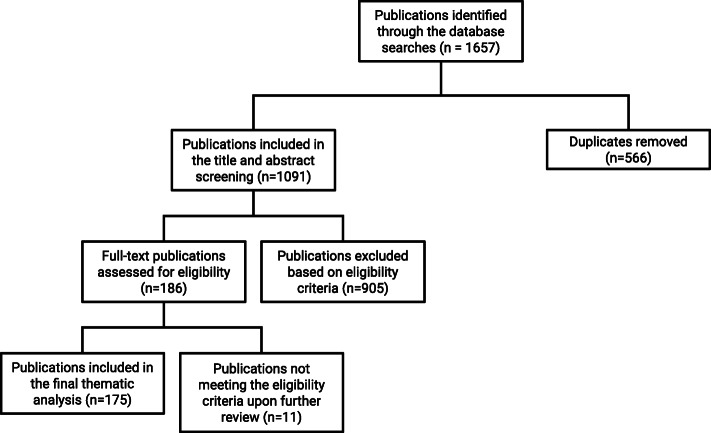
Flow chart of selection process

Arksey and O’Malley [8] note that scoping reviews are a relatively novel approach relative to the traditional approach of systematic literature reviews. In contrast to systematic literature reviews, which aim to synthesize and aggregate findings on a highly-focused research question, scoping reviews present an overview of broader topics typically encompassing a larger and more diverse body of literature [10]. Taking this distinction into account, the flexibility provided by a scoping review was determined to be invaluable for addressing the volume and diversity of literature, both academic and grey, on the integration of sex and gender considerations in health policymaking. A flow chart summarizing this search process can be found in Fig. 1. Adhering to the methodological framework outlined by Arksey and O’Malley [8], this scoping review employed the following five-stage model: (i) identifying the research question; (ii) identifying relevant studies; (iii) study selection; (iv) charting the data; and (v) collating, summarizing, and reporting the results.

### Stage i: Identifying the research question

This scoping review was guided by the following research question: ‘To what degree has sex and gender been inclusively and comprehensively used in health policymaking’?

### Stage ii: Identifying relevant studies

In an effort to include a wide breadth of literature of relative recency, our scoping review employed a 20-year time span, from January 2000 to June 2020. In order to capture as much of the relevant literature as possible, a broad search strategy was employed in consultation with a McMaster University librarian, inclusive of both the academic and the grey literature. The following nine databases were selected due to their perceived relevance to the research question: Academic OneFile, Canadian Public Documents Collection, EBSCO, LexisNexis, Policy and Society, ProQuest, Scholar Portal Journals, Studies on Women & Gender Abstracts, Web of Science. An individualized search strategy was tailored to each of these databases, created from some combination of boolean operators and the following 20-sex search terms: ‘checklist*’, ‘female’, ‘FTM’, ‘gender’, ‘genderqueer’, ‘gender aware*’, ‘gender divers*’, ‘gender mainstreaming’, ‘gender respons*’, ‘gender sensitiv*’, ‘intersex’, ‘male’, ‘men’, ‘MTF’, ‘non-binary’, ‘polic*’, ‘policy implementation’, ‘policy making’, ‘sex’, ‘standards development’, ‘technical standards’, ‘tool*’, ‘transgender*’, ‘transs*’, ‘two-spirit’, and ‘women’. The method yielded 1657 results across all databases.

### Stage iii: Study selection

As had been expected, this search strategy picked up a large number of irrelevant studies. A set of inclusion criteria was developed post hoc and then applied to all the citations yielded by this search strategy. Citations were included into the scoping review based on the following criteria: (i) publication date was between January 2000 and June 2020 inclusive; (ii) discussed some sort of metric for assessing the integration of sex and gender considerations in health policymaking; and (iii) was published in the English language. After duplicates were removed from the 1657 citations yielded by the initial search method, the reviewers applied the inclusion criteria to the titles and abstracts of the 1091 remaining citations. This method yielded 186 publications from the initial 1657 results. These publications were divided among the authors and read in full for subsequent stages of the scoping review.

### Stage iv: Charting the data

**Fig. 2:**
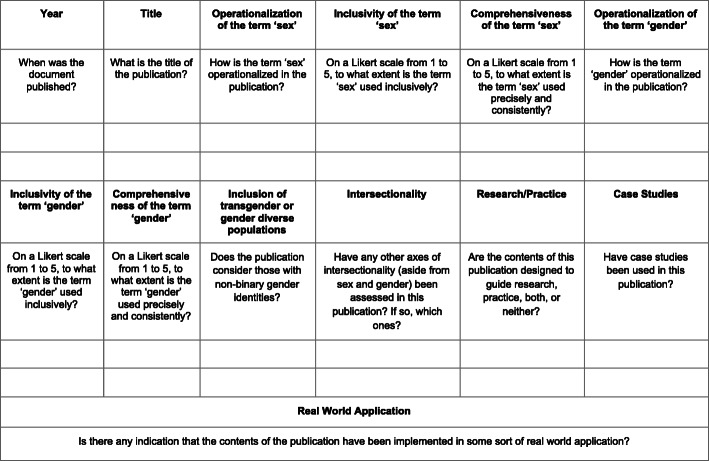
Categories used for charting the data from articles assessed in the scoping review

**Fig. 3:**
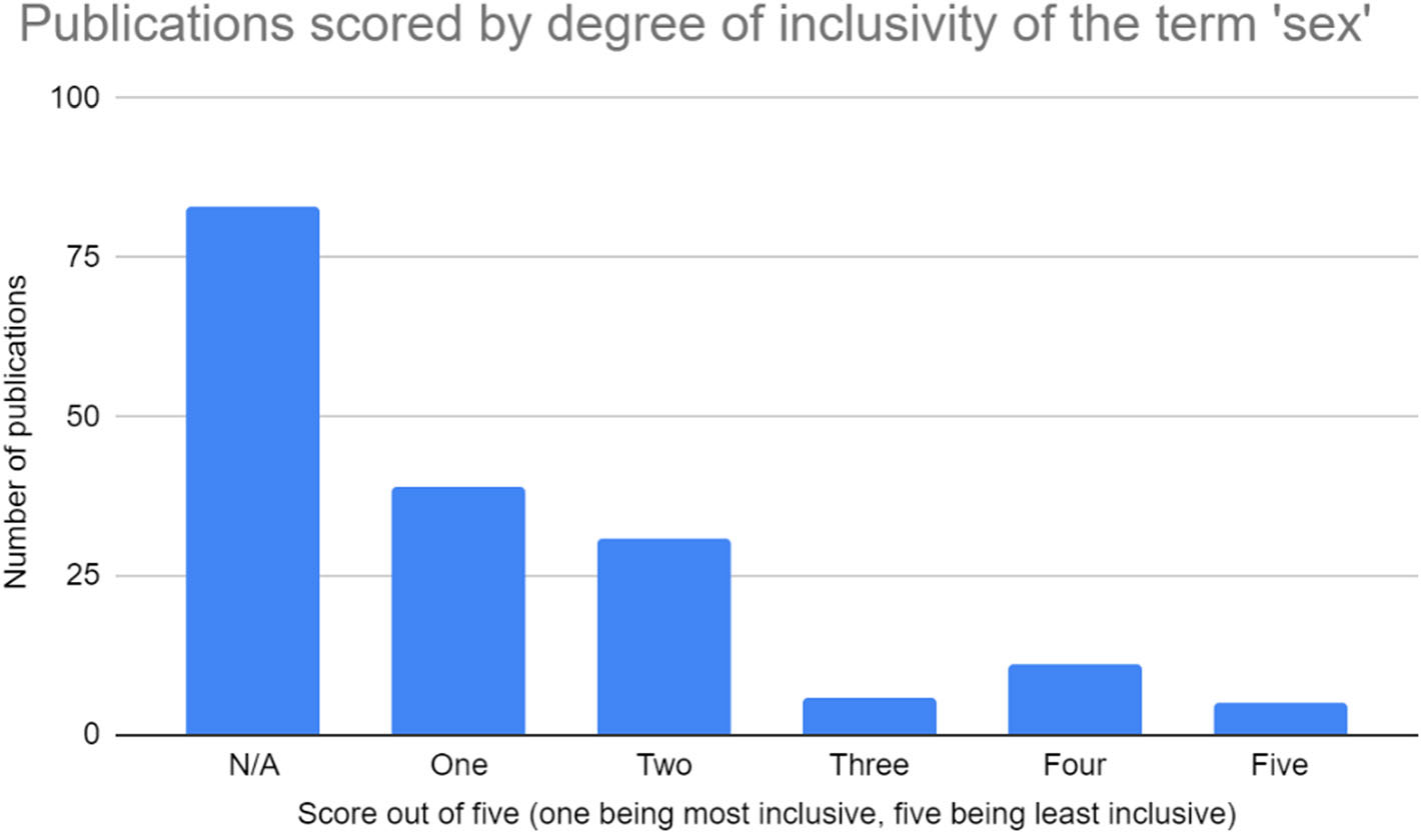
Publications scored based on their degree of inclusivity of the term ‘sex’

**Fig. 4:**
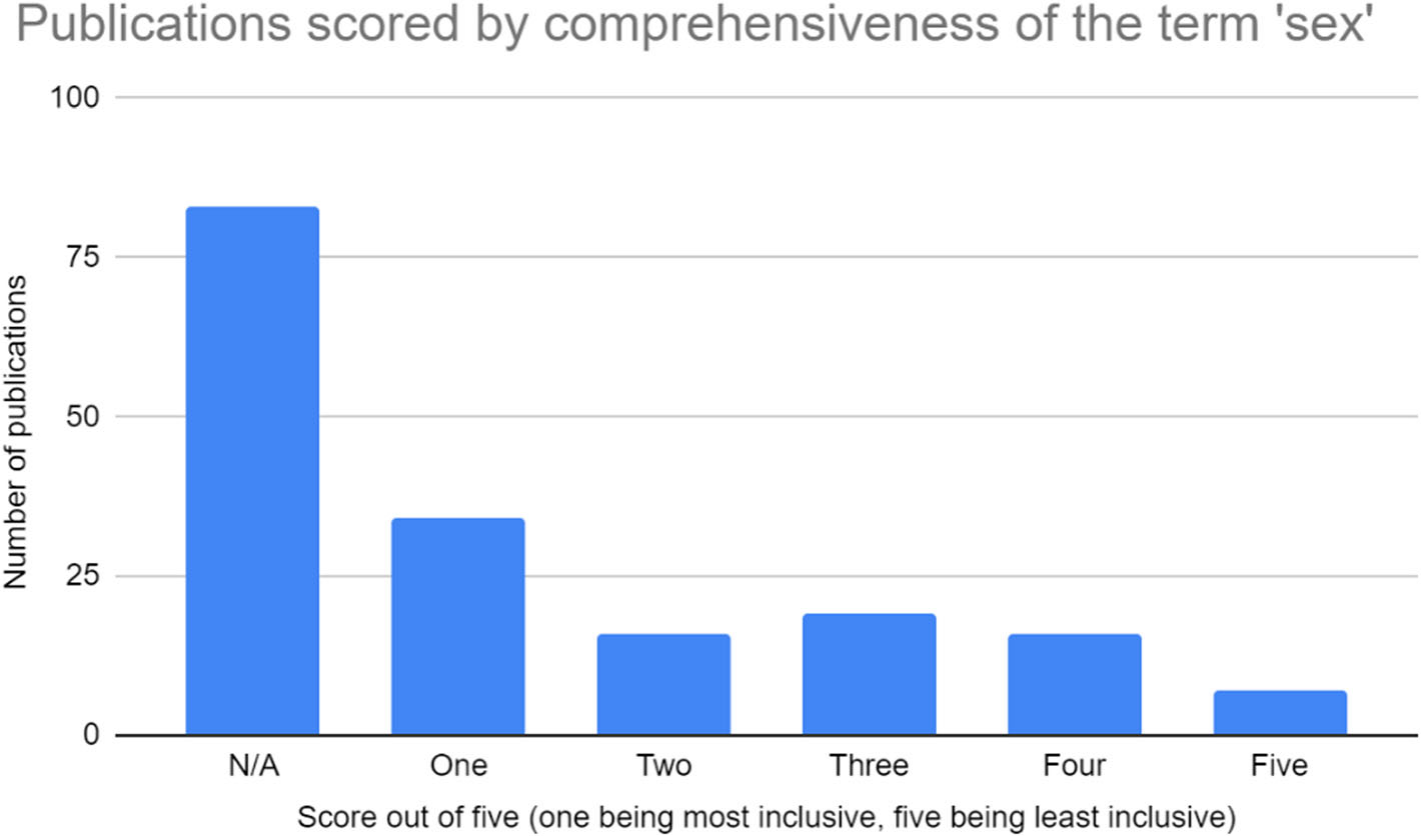
Publications scored based on their comprehensiveness of the term ‘sex’

**Fig. 5:**
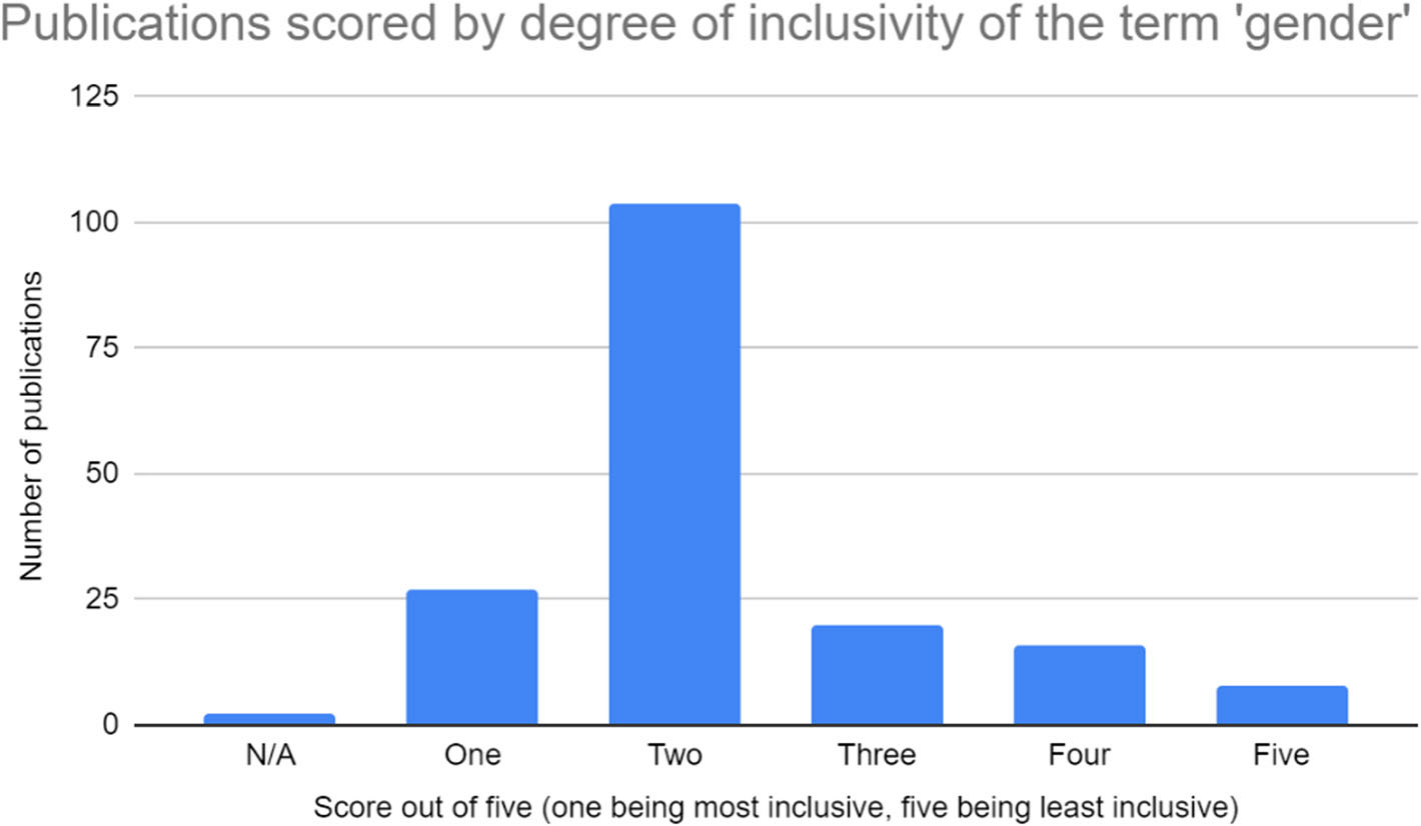
Publications scored based on their degree of inclusivity of the term ‘gender’

**Fig. 6:**
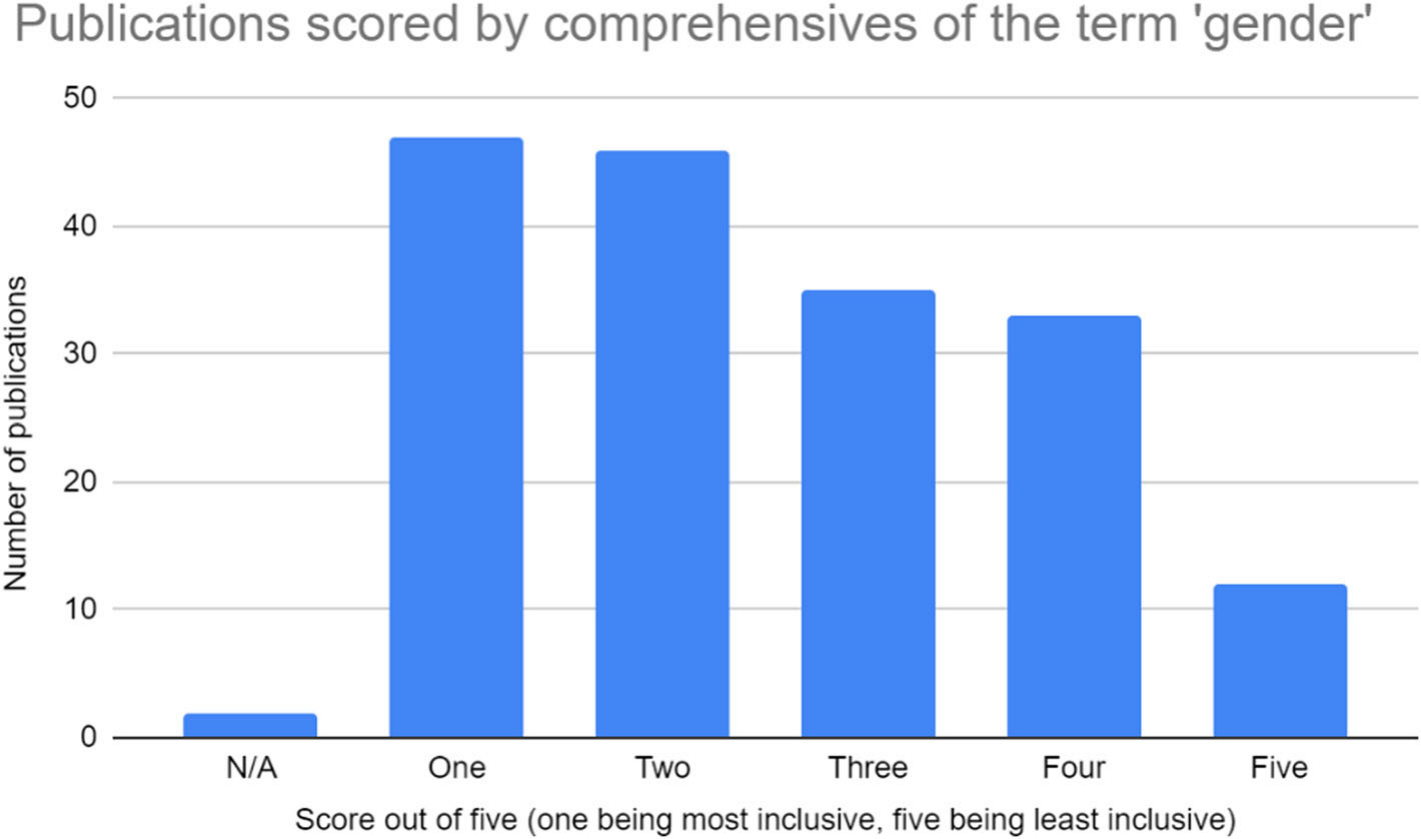
Publications scored based on their comprehensiveness of the term ‘gender’

All four authors participated in extracting and charting the data from the 186 publications [11–178] slated for inclusion. The key findings of these publications were documented on a spreadsheet available to all four authors. The following information was recorded from each publication, as outlined on Fig. 2 below: (i) year of publication; (ii) publication title; (iii) operationalization of the term sex; (iv) inclusivity of the term sex (scored using a five-point Likert scale, with one being least inclusive and five being most inclusive); (v) comprehensiveness of the term sex (scored using a five-point Likert scale, with one being least inclusive and five being most inclusive); (vi) operationalization of the term gender; (vii) inclusivity of the term gender (scored using a five-point Likert scale, with one being least inclusive and five being most inclusive); (viii) comprehensiveness of the term gender (scored using a five-point Likert scale, with one being least inclusive and five being most inclusive); (ix) inclusion of transgender or gender diverse populations; (x) consideration of other axes of intersectionality; (xi) relevance to research or practice; (xii) use of case studies; (xiii) indication of real world application. These categories have been summarized in Fig. 2. Furthermore, a graphic summary of the publications assessed in this review, as well as their corresponding scores on the aforementioned Likert scales, is provided in the bar graphs shown in Figs. 3, 4, 5, and 6.

In assessing the inclusivity and comprehensiveness of the documents’ use of sex and/or gender, attention was paid to if, and how, sex and gender were defined. If they were defined, the authors further determined: if this definition made a meaningful distinction between the terms; if this definition was used consistently, and whether the document recognized diversity within these groups.

As noted, sex and gender inclusivity were assessed using a five-point Likert scale which identified the extent to which documents recognized the existence of intersex and transgender populations, as well as recognition of the diverse experiences of individuals within sex and gender subgroups. Sex and gender comprehensiveness were also assessed using a five-point Likert scale which identified how the documents defined sex and gender, whether the definitions were distinct from each other, and whether these definitions were used comprehensively. The measures assessing comprehensiveness were added after it became apparent that many documents which defined sex and gender in their opening paragraphs as being distinct biopsychosocial phenomena would then go on to use the terms ‘man/woman’ and ‘male/female synonymously. For a document to receive the highest possible ranking on all measures, it would need to be: inclusive of intersex and transgender populations, recognize the diversity within the sex and gender categories used, distinctly and appropriately define sex and gender, and use the terms consistently and at no point synonymously.

### Stage v: Collating, summarizing, and reporting the results

While reading the full-text publications, 11 books were determined to fall outside of the study inclusion criteria, and were thus excluded from the final analysis. The final sample of 175 publications included both academic and grey literature. Approximately 36.9% of the publications included in this final sample were peer-reviewed academic articles. The vast majority (approximately 63.1%) of publications in the final sample were from the grey literature. Approximately 36.9% of these publications from the grey literature could be described as policy toolkits, intended to provide a rigorous and standardized framework to guide the policymaking of organizations. The remaining 63.1% could be described as policy papers, intended to contextualize policy issues for non-academic audiences, highlight current best practices in policy making, and provide broad recommendations to guide the policymaking of organizations. The grey literature was retrieved from a diverse array of geographical contexts, with government documents originating from international organizations, regional agencies, and national governments. The diverse origins of these documents, while unexpected, allowed for the application of a geographic lens to the results of the scoping review. A comprehensive summary of the results of the scoping review are presented below, in the ‘Results’ section of this paper.

## Results

As illustrated in the flow diagram in Fig. 1, nine databases were searched to extract 186 sources determined to meet the inclusion criteria. Books were removed from the total number of sources, leaving 175 sources. While this is a large number when compared to other knowledge synthesis approaches (i.e. systematic review), the authors choose to conduct a full review of each of these 175 sources. Of the 175 sources included in the scoping review, 43 were policymaking toolkits, 69 were policy or government documents, and 63 were academic peer-reviewed articles.

**Fig. 7:**
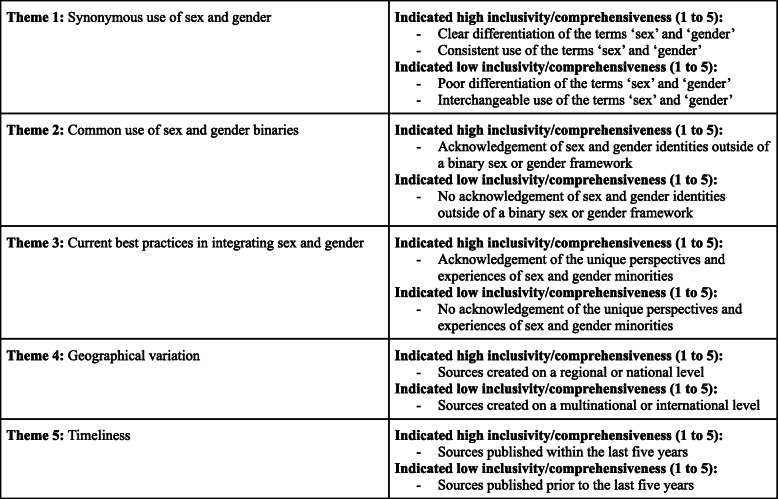
Summary of key findings relevant to each theme

Given our purpose of determining the extent to which sex and gender are accounted for in the policy process, the authors met weekly for 2 months to discuss the findings of their respective readings of the extracted sources. The original themes were determined after reading 50% of the extracted sources; these thematic results were confirmed and elaborated on further via the remaining sources: (1) synonymous use of gender and sex, with prioritization of gender; (2) common use of gender/ sex binaries; (3) current best practices in integrating sex and gender; (4) geographical variation in comprehensiveness, and; (4) timeliness, with documents published more recently being more comprehensive. The key findings have been summarized in Fig. 7.

### Synonymous use of gender and sex

The vast majority of sources used sex and gender synonymously, often prioritizing the use of gender to describe both sex and gender. This was evident in the United Nations Women’s *Progress of the World’s Women 2015–2016* report, in which the term gender was frequently used in reference to both “biological differences” and “socially-determined differences” [111]. In the few instances were sex was discussed, the term was similarly arbitrarily used in reference to both biological and social variables. Furthermore, the document made no distinction between the terms ‘male/female’ and ‘men/women’. The terms are used entirely interchangeably, sometimes even within the same sentence, further alluding to the document’s conflation of sex and gender [111]. This suggests an inadequate understanding of the distinction between these two terms.

The synonymous use of gender and sex also suggests a very constrained interpretation of these terms, often exclusive to the binary male/female or men/women dichotomies. In the vast majority of documents, gender was operationalized in terms of this binary framework (*n* = 129). As a result, these documents often failed to consider the perspectives of individuals who do not conform to the traditional masculine or feminine gender norms. This suggests that transgender, genderqueer, and third gender individuals are frequently excluded from the decision-making processes outlined by these documents, in turn reducing the efficacy of these processes as they apply to these gender minorities. Sex, when discussed, was often operationalized in a similar binary framework (*n* = 70). This frequently resulted in a lack of consideration for certain groups, namely intersex individuals, who do not conform to the binary male/female framework of sex.

Related to this is the common use of sex-disaggregated data being used as a proxy for gender outcomes. Sex-disaggregated data was presented in many sources as evidence of gender inequities. In such cases, the terms sex and gender were often assumed to be synonymous, and no effort had been made to acknowledge how this discrepancy had been taken into account. For instance, in the *Gender Mainstreaming in Practice* handbook published by the United Nations Development Programme, the author states that “all indicators should be disaggregated by sex wherever possible [as] this helps identify the gender differentiated impact of our interventions” [15]. In this instance, no attempt is made to consider the degree to which sex-disaggregated data may be of relevance to gender-oriented strategies.

Several documents offered alternatives to using sex-disaggregated data as a proxy for gender variables. For instance, the *Toolkit for a Gender-Responsive Process to Formulate and Implement National Adaptation Plans (NAPs)* lists ‘gender analyses’ as a distinct measure which may be used to assess gender dynamics and inequities [156]. The *Gender Mainstreaming Checklist for the Health Sector*, published by the African Development Group, similarly advocated for the use of ‘gender indicators’ to measure gender-related changes over time [47].

### Common use of sex and gender binaries

Related to the first theme, many sources used constrained definitions of sex and gender, which were often limited to a binary understanding. That is, populations were deemed to fit within one of two given categories, whether male/female in the case of sex, or women/men in the case of gender. Of the documents included in the scoping review, the majority used binary definitions of gender (*n* = 129) or sex (*n* = 153). As a result, populations which do not conform to the traditional, binary understanding of sex and gender were frequently excluded from consideration in these documents. The perspectives of intersex individuals were only considered alongside cis males and females in 22 of the documents, whereas the perspectives of transgender and gender diverse individuals were only alongside cis men and women in 41 of the documents.

Some plausible explanations for this include the relative under-representation of transgender populations in research in general [179], particularly outside of Canada and Northern Europe, the perceived difficulty of accounting for transgender populations in quantitative research [180, 181], and the widespread imposition of gender binaries during colonization, effectively erasing the strong pre-colonial histories of third gender and transgender populations in many Asian and South American countries [182, 183].

### Current best practices in integrating sex and gender

Of the documents which identified transgender and gender diverse populations as a subject of interest in SGA+ (*n* = 29), only a subset (*n* = 11) did so in a truly inclusive and comprehensive way. While several of these papers explicitly focused on transgender populations as their primary subject of study [30, 32, 38, 53, 92], some of the most inclusive and comprehensive papers approached transgender and gender diverse populations alongside cisgender populations as part of an overall approach to gender analysis. Articles that focused on HIV/AIDS or sexual violence, particularly within the global south, were typically more inclusive of transgender women as populations with higher exposures to risks associated with sexual violence and HIV/AIDS [33, 111, 149, 171], generally as a result of their over-representation within the commercial sex trade. Transgender men were rarely discussed except in the context of broader gender minorities, although some documents focusing on sexual and reproductive health note that this population is highly vulnerable to sexual violence [111, 149, 184–186].

It is important to note that while many documents addressed binary transgender populations, representation of non-binary and third genders were typically ignored. This is important to note as many Indigenous cultures, particularly in the global south, had strong traditions of third genders and non-binary genders that pre-date colonization. Documents which recognized this [147, 149] were typically focused on or produced by scholars working within the global south. Indeed, the document which was assessed to have the most comprehensive and inclusive approach to sex and gender, and which recognized the experiences of transgender populations as existing within the broader context of gendered oppression, was focused on the gendered differences in experiences of public transit in Pakistan [147]. This document included the experiences of cisgender men and women on public transit but also focused on the experiences of transgender Pakistanis, many of whom identified as being hirja, a third gender which was present throughout the Indian subcontinent and South Asia prior to colonization. By considering hirja experiences alongside the experiences of cisgender women and men, this document engages deeply in a comprehensive and inclusive gender analysis which highlights the diverse experiences of transgender people in the context of broader gendered experiences of public transit.

### Geographical variation

Given political structures and history, religious beliefs and cultural norms within particular societies across the world, certain geographical regions and nations were found to be comparatively more comprehensive and inclusive in their interpretation of sex and gender than others. For example, although there were comparatively fewer sources from, or representative of, countries within the regions of Southeast Asia and South America, those that were examined and which included a comprehensive approach to sex and gender were found to be more inclusive of non-binary and third-gender transgender populations [53, 149, 171], while the documents from Western Europe and North America which included transgender populations typically highlighted the experiences of binary transgender men and women [28, 32]. It should be noted that a number of Canadian documents also included two-spirit populations [50, 92], a distinct difference from the United States and Western Europe. It is also important to note that while a number of papers (*n* = 29) were inclusive of transgender populations, very few (*n* = 8) included intersex populations, meaning that a number of the documents which took a comprehensive and inclusive approach to gender did not extend the same level of inclusivity to sex.

The extracted sources, in totality, illustrated that regional or nationally produced sources were more likely to have enhanced comprehensiveness when compared to those sources created by a grouping of nation states, such as those sources produced by the transnational organizations, for example. Documents produced for international bodies, particularly within the grey literature, typically defaulted to more conservative and less comprehensive approaches to sex and gender. The exception to this general trend was found in papers produced for International NGOs dedicated to sexual and reproductive health, which were more comprehensive [149, 171].

### Timeliness

The passage of time has proven to stretch the understanding of both sex and gender as distinctive continuums, rather than restrained categories. In contrast, many sources published in the early 2000s used a much more confined interpretation, with sex and gender being viewed as binary measures which were regarded to be interchangeable with one another. Sources more recently published in the last 5 years were more likely to be comprehensive and inclusive than those published closer to the beginning of the time period of concern. The majority of these recent sources were from the grey literature (*n* = 54), although several were found in the peer-reviewed academic literature (*n* = 31). This reflects an expansion of knowledge and understanding of both sex and gender, and suggests a greater acceptance of sex and gender as unique continuums, and both as relevant axis’ of diversity. The ‘Gender Assessment Tool’ of the International Planned Parenthood Federation, published in 2019, is illustrative of this [171]. The authors of the document clearly define gender as “a fluid concept that is present through all social life” which “is not biological or natural but is constructed from the images, messages and expectations we see around us”, and emphasize that the concept is distinct from sex, which is said to consist of “biological features, such as male or female anatomy” [171]. If this trend were to continue, as expected, the passage of time will see an increase in comprehensibility and inclusivity of both sex and gender.

### Limitations

Recognizing the rigorous search strategy informed by a university librarian, there are two limitations evident. Given the time-intense process of reading each document, each document was reviewed by only one of the four authors. Documents would have been more thoroughly reviewed if more than one author was involved, ideally providing a cross-check. Further, English-language sources were solely considered for inclusion. As a result, it is possible the review may have missed relevant documents written in other languages.

## Conclusion

There has been a recognition by researchers and policymakers, both government and non-governmental national and international organizations, of the need to establish and promote sex and gender equality, especially with respect to accessing appropriate health opportunities. The objective of this scoping review was to determine the inclusivity and comprehensiveness of the use of sex and gender in health policymaking. The process consisted of a review of existing literature, both grey (*n* = 110) and academic (*n* = 65), to identify prior instances of the use of sex and gender in health policymaking. These were used to determine gaps and assess best practices. The results of the scoping review have shown that while the call to action has grown over the years, there has been a slow adoption to integrate a more comprehensive definition of sex and gender in health policymaking.

In order to lay a foundation for this study, there was a need to establish a definition and broad operationalization of the terms sex and gender. The distinction between these two terms is critical given our interest in developing an inclusive and comprehensive approach that fills the current gap in the assessment of sex and gender considerations in health policymaking. Certain themes emerged from the literature. First, while sex is defined as the genetic, physiological, and biological characteristics that distinguish males and females, and gender refers to the socially constructed characteristics that distinguish men and women, it was found that the vast majority of sources used the two terms synonymously, often prioritizing gender to describe both. This implied a narrow interpretation of these terms, leading to a simple binary view of women and men and, consequently, resulting in an inadequate understanding of each term’s distinctiveness. Our study identified this as a major gap. In order to narrow or eliminate this gap, toolkits need to develop metrics that make the distinction between sex and gender and expand their definitions to be more inclusive of transgender and non binary lifestyles.

The second theme is closely related to the first. Our study was particular in reviewing the inclusivity and comprehensiveness of the definitions of sex and gender. Inclusivity was defined as the extent to which the paper took into account persons outside of the traditional simple binary view of women and men, as well as the heterogeneity of individuals beyond sex and gender. Comprehensiveness was measured by how consistently and accurately the terms were used in the publication. The study revealed that transgender and other gender diverse populations were rarely included in these publications. Although plausible explanations may be those of simple under representation of transgender populations in the research, and/or the colonizing impact of imposing a simple binary viewpoint, it still remains that in the timeframe from the year 2000 on, there were few publications that made the effort to include transgender and other gender diverse populations. Our study identified this as a secondary gap. In order to have the most holistic and inclusive health policy for all, there has to be the recognition that the definition of sex needs to be expanded to include transgender and non binary persons and that sex is not to be used synonymously with the term gender but is to be considered on its own merits and operationalized by metrics that take this into account.

Fourthly, it was found that certain geographical regions and nations were more inclusive and comprehensive in their interpretation of sex and gender. Regions of Southeast Asia and South America were more inclusive of non-binary and third-gender transgender populations, while papers from North America and Western Europe, which included transgender populations, typically only highlighted experiences of binary transgender men and women. That being said, there were several noteworthy exceptions to these regions, most notably in the documents from Canada and the Nordic Countries. It was noted that few publications included intersex populations. In any case, those papers that took a comprehensive and inclusive approach to gender did not usually extend that thoroughness to sex. Generally, regional and nationally produced sources were more comprehensive in their studies, while studies from international organizations were more conservative and less comprehensive in their approach to sex and gender. The limitation of only reviewing publications in English may have prevented a more expansive scoping review. The limitation of only reviewing publications in English may have prevented a more expansive scoping review. This limitation is more difficult to overcome without the ability to speak another language. However, it is possible to have articles translated from other countries to enhance the thoroughness of any future review. It is possible that greater strides have already been taken to have a more inclusive and comprehensive approach to health policy, but they are simply not available in the English language, and it behooves us to reach out to find out if better practices exist out there.

Finally, it was found that publications in the last 5 years were more inclusive and comprehensive of sex and gender than those published earlier. This indicates more recent awareness and understanding, as well as a comparative increase in knowledge regarding the acceptance and importance of expanding the inclusivity and comprehensiveness of sex and gender. This scoping review highlights the need for policymakers to: enhance their ability to better incorporate transgender and other diverse populations via a more inclusive and comprehensive understanding of sex and gender; develop a more holistic approach to health policymaking, and consequently; provide better health policymaking outcomes. This is critical if our health policies are to be inclusive of everyone they serve. A better understanding of the term sex can lead to a better ability to address the special needs of women, men, transgender and non binary persons, alike. This, with a better understanding of the term gender, will bring the balance necessary to make our health policy the most inclusive and comprehensive one it can be.

This paper, and many of the most comprehensive and inclusive articles, understands sex and gender as representing important determinants of health which must be understood in the context of other social determinants of health. Comprehensive and inclusive approaches to gender and sex often also considered the relevance of socioeconomic status, racial or ethnic identity, religion, age, and migration status, suggesting that a comprehensive approach to sex and gender also often entails a more comprehensive approach to other aspects of identity and health.

It is important to note that there are real-world consequences when policy documents fail to address sex and gender in a comprehensive and inclusive way. All of the papers which were assessed as failing to adequately operationalize comprehensive understandings of sex and gender failed to account for transgender, gender diverse, and intersex populations. This is meaningful: the erasure of transgender and gender diverse populations has been linked with poorer health outcomes for transgender patients [179, 180]. Further, the failure to adequately distinguish between sex and gender has scientific implications beyond the political and social realms. Transgender women are often at a higher risk of HIV transmission due to their overrepresentation in the commercial sex trade, but studies on HIV transmission typically either exclude them from consideration or conflate them with men who have sex with men (MSM) [181]. This limits the ability of researchers and policymakers to understand the social and behavioural factors which increase HIV vulnerability among trans women and may lead to potential inadequacies in HIV prevention policies based on this research.

In summary, this scoping review revealed clear gaps in the inclusive and comprehensive incorporation of sex and gender in the development of health policy. It showcased the need for new standards, tools and strategies that could overcome these gaps. This scoping review has provided the foundation to inform a sex and gender inclusive toolkit for policymakers, which would better integrate sex and gender considerations in a more comprehensive and holistic approach to health policymaking. Following the methodological framework outlined in Day et al. (7)*,* next steps need to be taken to implement the findings from this scoping review to develop metrics that policymakers can use to create best policy that best integrates sex and gender considerations. The next steps would follow those outlined in Day et al. (7). These steps include seeking input from select study participants (i.e. those involved in health policy development), by way of a questionnaire reviewing a draft set of metrics integrating sex and gender in health policy. The input will help refine the set of metrics and, ultimately increase the metric’s credibility for use in the health policy field.

## Data Availability

Data is available upon request from the corresponding author.
